# Nutritional Outcomes of Overdentures vs. Complete Dentures in Older Edentulous Adults: A Systematic Review and Meta‐Analysis

**DOI:** 10.1111/joor.70111

**Published:** 2025-12-16

**Authors:** Danielle Soley Batista, Gabriela Aparecida Winkert Manfron, Diulia Pereira Bubna, Débora Marta Barbosa, José Stechman‐Neto, Karinna Veríssimo Meira Taveira, Cristiano Miranda de Araujo, Flávio Magno Gonçalves, Thalita de Paris Matos Bronholo

**Affiliations:** ^1^ School of Dentistry Tuiuti University of Paraná Curitiba Paraná Brazil; ^2^ Postgraduate Program Dentistry and Health of Human Comunnication Tuiuti University of Paraná Curitiba Brazil; ^3^ Postgraduate Program in Speech, Language and Hearing Sciences Federal University of Rio Grande Do Norte Natal Brazil; ^4^ Postgraduate Program in Communication Disorders Tuiuti University of Paraná Curitiba Brazil; ^5^ Postgraduate Program Dentistry Tuiuti University of Paraná Curitiba Paraná Brazil; ^6^ School of Dentistry Federal University of Paraná Curitiba Paraná Brazil

**Keywords:** complete dentures, edentulism, implant‐supported overdentures, meta‐analysis, nutrition, systematic review

## Abstract

**Objective:**

To investigate whether implant‐supported overdentures provide nutritional advantages compared with conventional complete dentures in older edentulous adults, through a systematic review and meta‐analysis.

**Methods:**

A comprehensive search was performed in PubMed/MEDLINE, Scopus, Web of Science, Cochrane Library, EMBASE, LILACS, LIVIVO and grey literature sources. Eligible studies included completely edentulous patients aged ≥ 60 years rehabilitated with overdentures or conventional complete dentures, with nutritional intake assessed using validated methods. Data extraction and risk of bias assessments were conducted independently by calibrated reviewers. Random‐effects meta‐analyses were performed, and the certainty of evidence was rated using GRADE.

**Results:**

Nine studies met the inclusion criteria, of which five were included in the quantitative synthesis. Overdenture users showed significantly higher vitamin B12 levels at 6‐month follow‐up (SMD = 0.60; 95% CI: 0.18–1.02; I^2^ = 54%), but no consistent differences were observed for albumin or folate. Overall certainty of the evidence was rated as moderate for vitamin B12 and albumin, and low for folate, due to methodological limitations, heterogeneity in outcome measures and small sample sizes.

**Conclusion:**

While overdentures improve masticatory function and may transiently enhance vitamin B12 status, the current body of evidence does not support a consistent nutritional advantage over conventional dentures. High‐quality, long‐term trials are needed to clarify the systemic nutritional implications of prosthetic rehabilitation in older adults.

## Graphical abstract

The Challenge of Edentulism: Older edentulous adults often face significant difficulties in chewing, leading to restrictions in their diet and potentially affecting their overall nutritional status. Rehabilitation Options: Two primary prosthetic rehabilitation methods were compared: conventional complete dentures and implant‐supported overdentures, aiming to restore masticatory function. Nutritional Outcomes: A systematic review and meta‐analysis of biochemical markers revealed that while overdentures may offer masticatory improvements, the nutritional benefits (e.g., albumin, folate) were not consistently superior to conventional dentures. A transient increase in Vitamin B12 was observed in the overdenture group at 6 months. Key Clinical Takeaway: Restoring chewing function through prosthetic rehabilitation alone is often insufficient to guarantee improved nutritional status. An interdisciplinary approach, integrating prosthetic treatment with professional nutritional counselling, is vital for optimising the health and well‐being of older edentulous patients.

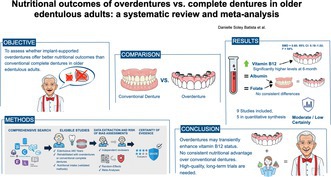

## 1 Introduction

Edentulism, defined as the complete loss of teeth in one or both arches, affects approximately 15.4% of the Brazilian population, with a higher prevalence among individuals aged over 60 years [1]. This condition is associated with various functional impairments, including compromised mastication, aesthetic concerns and speech difficulties, which can negatively influence dietary intake and nutritional status. These changes have systemic implications, such as reduced lean body mass and increased risk of nutritional deficiencies—factors strongly associated with chronic diseases and cognitive decline in older adults [2]. Biochemical analyses have shown that edentulous individuals tend to consume fewer vegetables, fruits and fibres compared to those with natural dentition [3].

Oral rehabilitation plays a central role in restoring function and improving the overall health and quality of life of edentulous patients. Evidence indicates that prosthetic rehabilitation is linked to improved nutrition and reduced mortality in older adults [4, 5]. Furthermore, patients rehabilitated with prostheses often report enhanced quality of life and a greater willingness to engage in healthier behaviours, especially when the prosthesis is well accepted and functionally satisfactory [5, 6].

Among the available treatment modalities, conventional complete dentures (CDs) are the most commonly used due to their lower cost, non‐invasive nature and satisfactory aesthetic results. However, they often lack adequate stability and retention, particularly during the mastication of harder foods, which may compromise dietary choices and lead to nutritional deficits. Implant‐supported overdentures (IODs), by contrast, offer improved stability and retention by anchoring the prosthesis to the alveolar bone, reducing bone resorption and enhancing masticatory performance [7]. Nevertheless, IODs require surgical intervention, entail a postoperative recovery period and are associated with higher financial costs, making them less accessible to some populations [8].

Accordingly, the aim of this systematic review and meta‐analysis was to evaluate whether nutritional intake differs between edentulous older adults rehabilitated with conventional complete dentures versus implant‐supported overdentures, based on the most current and comprehensive evidence available.

## 2 Material and Methods

This systematic review was conducted in accordance with the *Preferred Reporting Items for Systematic Reviews and Meta‐Analyses* (PRISMA) guidelines [9] and was prospectively registered in the *International Prospective Register of Systematic Reviews* (PROSPERO). The registration number is CRD42023474621.

### 2.1 Eligibility Criteria

The Inclusion and Exclusion Criteria Were Established Using the PICOS Framework, as Follows:

- **Population (P):** Edentulous individuals aged 60 years or older
- **Intervention (I):** Oral rehabilitation with implant‐supported overdentures
- **Comparison (C):** Rehabilitation with conventional complete dentures
- **Outcomes (O):** Nutritional intake assessed using validated methods
- **Study design (S):** Interventional and observational studies

### 2.2 Inclusion Criteria

Studies Were Eligible If They:

- Included completely edentulous patients aged ≥ 60 years;
- Compared nutritional outcomes between patients rehabilitated with conventional complete dentures and those with overdentures;
- Assessed nutritional intake using validated dietary or biochemical methods.

### 2.3 Exclusion Criteria

Studies Were Excluded If They:

- Included partially edentulous patients or individuals without prostheses;
- Did not include a direct comparison between complete dentures and overdentures;
- Were reviews, opinion pieces, in vitro or animal studies, conference abstracts, case reports or case series;
- Failed to report nutritional outcomes;
- Included patients younger than 60 years of age.

### 2.4 Information Sources and Search Strategy

A comprehensive search strategy was developed using a combination of controlled vocabulary (MeSH terms), free‐text terms and Boolean operators. Searches were performed in seven electronic databases: PubMed/MEDLINE, Scopus, Web of Science, Cochrane Library, EMBASE, Latin American and Caribbean Health Sciences Literature (LILACS) and LIVIVO. Grey literature was searched via Google Scholar and ProQuest.

All searches were conducted on 16 August 2023. Additionally, a manual search of the reference lists of included articles was performed using the Citation Chaser tool. Expert consultation was conducted to identify any potentially relevant unpublished or ongoing studies. The full search strategy is detailed in Appendix 1.

### 2.5 Study Selection

Two reviewers independently screened titles and abstracts for potential eligibility. Before full screening, a calibration exercise was performed involving 100 articles to ensure reviewer consistency. Inter‐rater agreement was assessed using Cohen's Kappa coefficient (κ), with a value > 0.8 deemed acceptable to proceed.

Screening Was Conducted in Two Phases:

1. **Phase 1**: Title and abstract screening.
2. **Phase 2**: Full‐text assessment for final eligibility.

The Rayyan platform (*Rayyan Intelligent Systematic Review*) was used to manage citations and facilitate independent, blinded screening.

Disagreements between reviewers were resolved through discussion or adjudication by a third reviewer.

### 2.6 Data Extraction

Two Reviewers Independently Extracted Data Using a Pretested Data Collection Form. Extracted Information Included:

- **Study characteristics**: authorship, year, country and study design;
- **Population data**: sample size, sex, mean age;
- **Intervention and comparator**: type of prosthetic rehabilitation;
- **Outcomes**: nutritional intake measures and assessment methods;
- **Follow‐up duration** and study conclusions.

Discrepancies were resolved by discussion and consensus.

### 2.7 Data Items

For Quantitative Synthesis, the Following Were Extracted:

- Mean and standard deviation (SD) of nutritional variables;
- Sample sizes per group;
- Follow‐up time post‐intervention (in months).

When SDs were not directly available, they were estimated using reported confidence intervals or other dispersion metrics.

### 2.8 Risk of Bias Assessment

Risk of Bias in Observational Studies Was Assessed Using the **JBI Critical Appraisal Tools (2020)** [10], Applying the Checklist Appropriate to Each Study design. Each Domain was Classified as ‘Yes’, ‘No’, ‘Unclear’ or ‘Not Applicable’. An Overall Risk Classification was Assigned as Follows:

- **Low risk**: > 70% ‘Yes’ responses;
- **Moderate risk**: 50%–69%;
- **High risk**: < 50%.

For randomised controlled trials (RCTs), the **Cochrane Risk of Bias Tool, version 2.0 (RoB 2)** was applied. This tool evaluates five domains comprising 17 signalling questions, categorised as ‘Yes’, ‘Probably Yes’, ‘Probably No’ or ‘No’. RCTs were classified as ‘Low risk’, ‘Some concerns’ or ‘High risk’ of bias.

### 2.9 Effect Measures

When outcome data were reported using different scales across studies, **Standardised Mean Differences (SMD)** were used to allow pooling in meta‐analysis. This enabled the integration of results into a unified effect size regardless of the original scale.

### 2.10 Synthesis Methods

Random‐effects meta‐analyses were performed using the **inverse variance method**, with heterogeneity quantified by the **I** ^**2**^ **statistic** and between‐study variance estimated using the **DerSimonian and Laird method** (Tau^2^).

Statistical significance was set at *p* < 0.05, with 95% confidence intervals (CI). All analyses were conducted in **R (RStudio v1.2.1335, Boston, USA)**, and forest plots were generated accordingly.

### 2.11 Assessment of Certainty in Evidence

The Certainty of Evidence for Each Outcome Was Assessed Using the **GRADE Approach (Grading of Recommendations Assessment, Development and Evaluation)** [11, 12]. The Following Domains Were Evaluated:

- Risk of bias
- Inconsistency
- Indirectness
- Imprecision
- Publication bias

Two reviewers (DSB and FMG) conducted the GRADE assessments independently. In case of disagreement, consensus was sought, with arbitration by a third reviewer (TPMB) when necessary. The certainty of evidence was classified as ‘High’, ‘Moderate’, ‘Low’ or ‘Very low’.

## 3 Results

### 3.1 Study Selection

**FIGURE 1:**
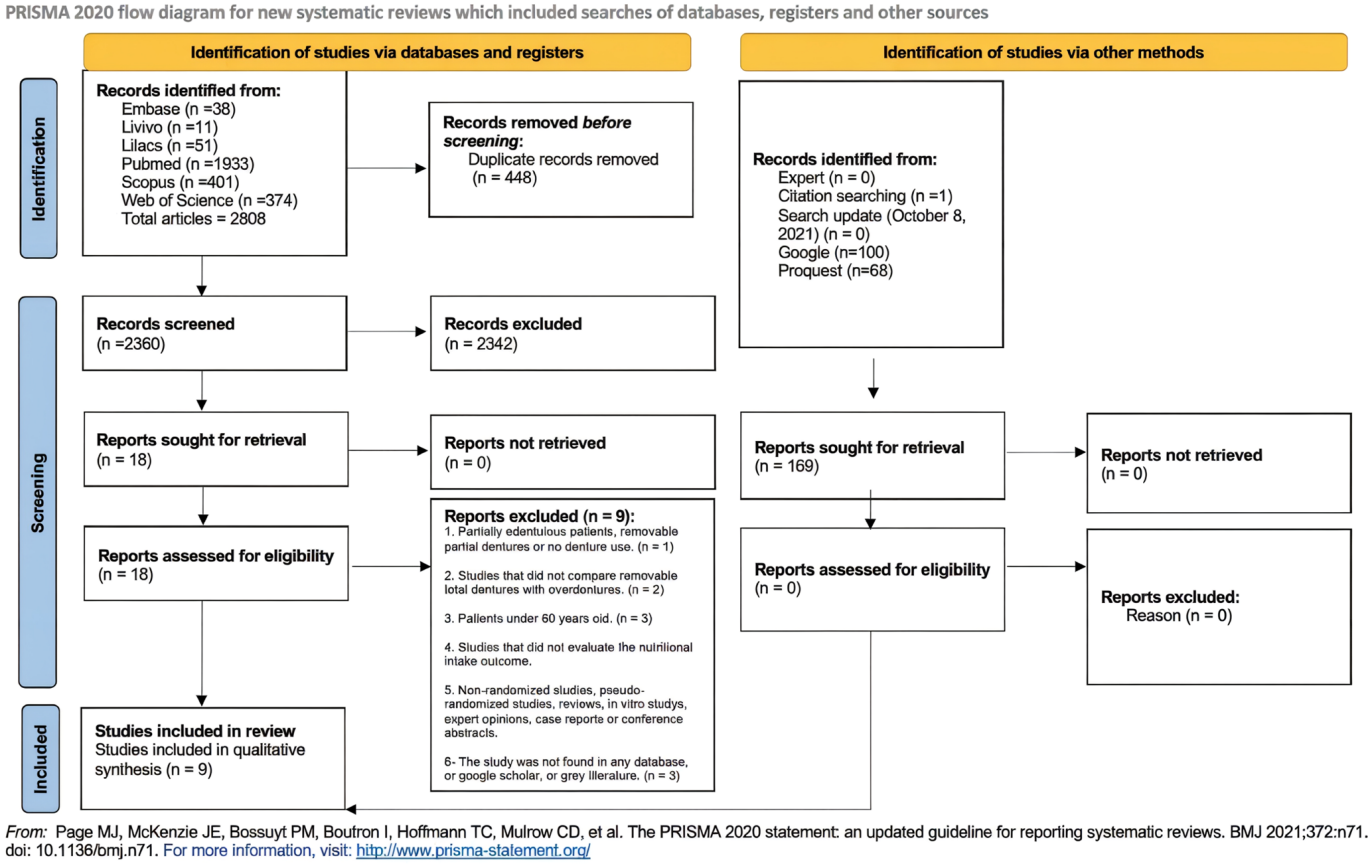
Flowchart of the study selection process.

A total of 2808 records were retrieved from the electronic and grey literature searches. After removing duplicates, 2360 references remained for screening. Of these, 18 full‐text articles were assessed for eligibility in Phase 2, with 9 studies excluded for not meeting the inclusion criteria (see Appendix 2). No additional studies were identified through manual search or expert consultation. The complete selection process is illustrated in Figure 1.

### 3.2 Study Characteristics

Nine studies met the eligibility criteria: three cohort studies [13, 14, 15], one cross‐sectional study [6] and five randomised controlled trials (RCTs) [16, 17, 18, 19, 20]. All cohort and cross‐sectional studies were conducted in Brazil [6, 13, 14, 15]. The RCTs were conducted in Canada [16, 18, 19], Norway [17] and Switzerland [20]. Assessment tools varied across studies and included: food diaries; the Mini Nutritional Assessment (Guigoz test); anthropometric measurements; biochemical parameters (e.g., serum vitamin and protein levels).

### 3.3 Participant Characteristics

Age range: 60 to 88 years; Sample sizes: 12 to 255 individuals; Both genders were represented, with a predominance of female participants.

### 3.4 Risk of Bias in Included Studies

**FIGURE 2:**
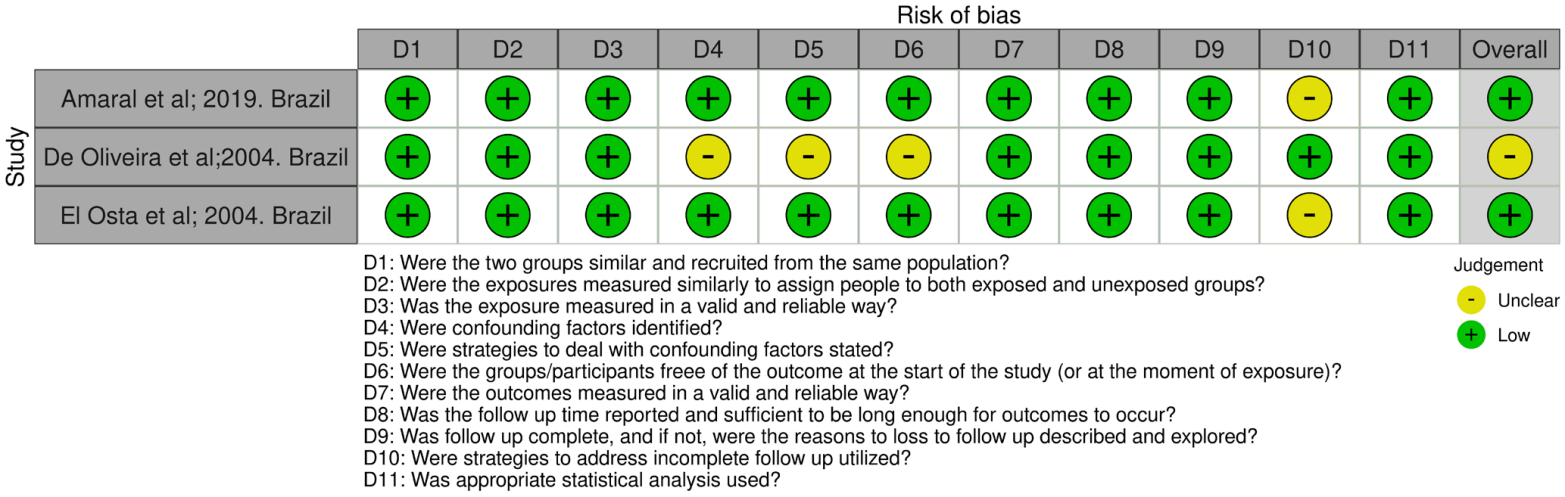
Risk of bias in the cohort studies.

**FIGURE 3:**
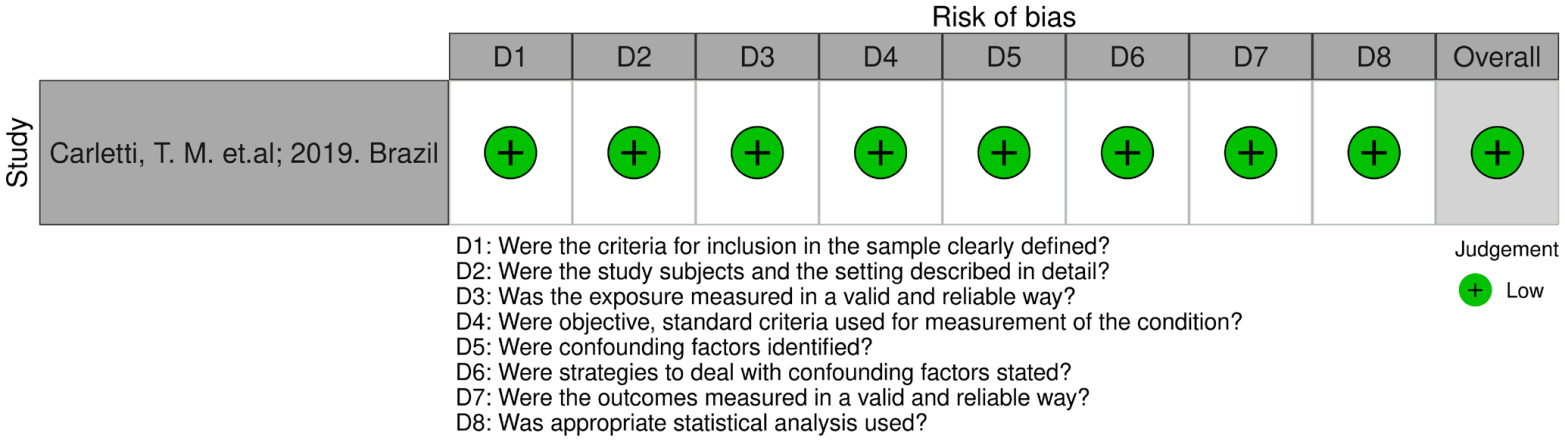
Risk of bias in the cross‐sectional study.

Among observational studies, only one [14] was judged to have a moderate risk of bias, primarily due to the lack of identification and control for confounding factors (Figures 2 and 3).

**FIGURE 4:**
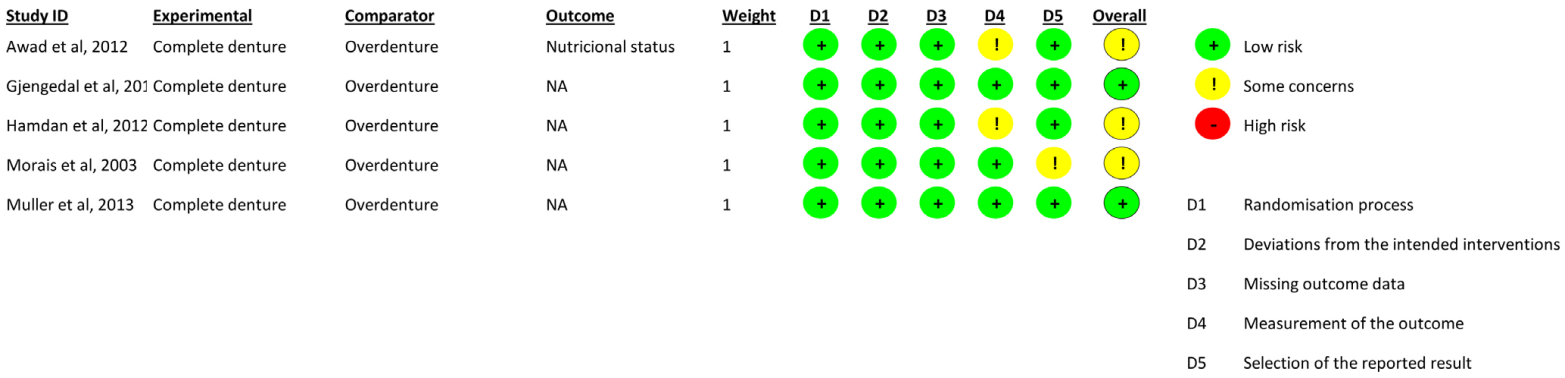
Risk of bias in the randomised clinical trials.

Of the five randomised clinical trials: three studies were rated as having a moderate risk of bias [16, 18, 19]; two studies were assessed as having a low risk of bias [17, 20]. Factors contributing to increased risk included: incomplete reporting of randomisation procedures; absence of trial registration protocols; ambiguity in outcome assessment (Figure 4).

### 3.5 Individual Study Results

#### 3.5.1 Nutrient Intake

The nutritional assessments in the included studies were conducted at baseline and follow‐up periods ranging from 3 to 12 months (Table 1).

**TABLE 1:** Characteristics of the selected studies.

Author, year and country	Study design	Sample (Gender)	Age	Evaluation time	Questionnaire/Tests
Amaral et al., 2019, Brazil	Cohort	12 (M = 4; F = 8)	+ 60	2–3 months	Jaw‐tracking kinesiographic device; Nutritional intake through a food diary.
Awad, MA, et al., 2012, Canada	Randomised clinical trial	255: Complete denture 127 (F = 71; M = 57); Overdenture—128 (F = 70; M = 57)	+ 65	6–12 months	Venous blood sample, Likert scale questionnaire.
Carletti, TM, et al., 2019, Brazil	Cross‐sectional	22: Overdenture—11 (n.r.); Complete denture—11 (n.r.)	+ 60	6 months	Dietary records over a period of 3 days; Dietbox programme.
de Oliveira, TR, et al., 2004, Brazil	Cohort	40: Complete denture—23 (n.r.); Overdenture—17 (n.r)	Elderly people	1 Year	Mini Nutritional Assessment (Guigoz test), interviews.
Gjengedal, H, et al., 2012, Norway	Randomised clinical trial	60: Complete denture—30 (n.r.); Overdenture—30 (n.r)	+ 76	2 years	Dietary records by telephone (Tuesday to Friday) in the 4, 8 and 11 months (questionnaire structured).
Hamdan, NM, et.al, 2013, Canada	Randomised clinical trial	217: Complete denture—114 (F = 71; M = 57); Overdenture—103 (F = 70; M = 57)	+ 65	Before the treatment and 12 months	3 days, 24‐h dietary records, at the beginning and 12 months after.
Morais, JA, et al., 2003, Canada	Randomised clinical trial	60: Complete denture – 30 (n.r); Overdenture—30 (n.r.)	65–75	Before treatment and 6 and 12 months after	3 days of dietary records, blood parameters and anthropometric measurements collected at the beginning of the study, at 6 and 12 months after.
Muller, F, et al., 2013, Switzerland	Randomised clinical trial	34: Complete denture—18 (F = 14; M = 4); Overdenture—16 (F = 9; M = 7)	+ 75 years	3–12 months	Body mass index, Mini Nutritional Assessment, blood markers and chewing efficiency with chewing gum.
Osta El, Nada, et al., 2017, Lebanon	Cohort	51 (M = 28; F = 23)	+ 60	Before treatment, 2–3 weeks after treatment, 3 and 6 months	Questionnaires, clinical examination, Mini Nutritional Assessment and GOHAI index.

Abbreviations: F: female; M: male; N.r: no related.

Key findings included: One study [13] reported a significant reduction in sodium intake and an increase in iron consumption among overdenture users. Another study [6] found that complete denture users had higher sodium intake, while overdenture users had elevated levels of vitamins B2 and B6. Despite these findings, both groups exhibited diets characterised by high caloric content and low nutritional quality, with deficiencies in magnesium, potassium, calcium, selenium, zinc, proteins, iron and vitamins.

#### 3.5.2 Patient Satisfaction and Eating Habits

Patients rehabilitated with overdentures reported higher levels of satisfaction compared to those using complete dentures, who expressed more dissatisfaction [6]. Improvements in masticatory performance were noted in the overdenture group, particularly in relation to the consumption of hard foods. However, both groups continued to exhibit low intake of essential vitamins and folate [16].

#### 3.5.3 Nutritional Status

Overdenture users generally demonstrated better nutritional status and a lower risk of malnutrition than complete denture users [14, 15]. Although overdenture users consumed fewer nutrients, their anthropometric parameters were more favourable, suggesting reduced vulnerability to malnutrition.

#### 3.5.4 Masticatory Ability

Rehabilitation with overdentures was associated with improved masticatory function, as evidenced by a reduction in food avoidance and an expanded dietary repertoire. However, there were no significant changes in body mass, and dietary records did not reveal differences between groups regarding energy intake or macronutrients [17].

#### 3.5.5 Macronutrient Intake and Body Composition

Comparative analyses showed: Higher intake of proteins, fats, carbohydrates, vitamins and folate among complete denture users; overdenture users had higher levels of riboflavin and protein, but lower intake of folate and total energy; regarding body composition, overdenture users exhibited higher body mass index (BMI) and elevated albumin, folic acid and haemoglobin levels. In contrast, C‐reactive protein was higher in the complete denture group [18, 20].

### 3.6 Quantitative Synthesis

**FIGURE 5:**
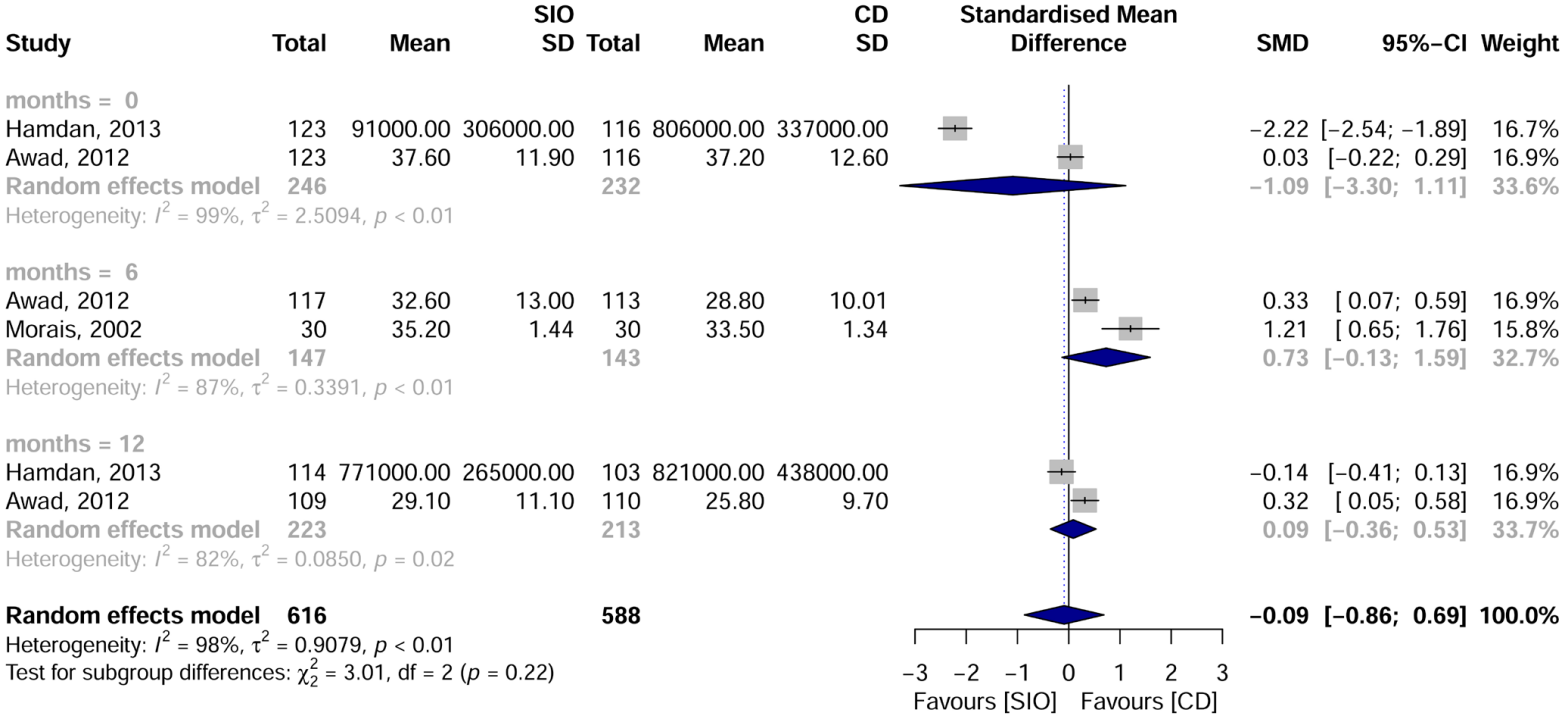
Meta‐analysis results for vitamin B12.

**FIGURE 6:**
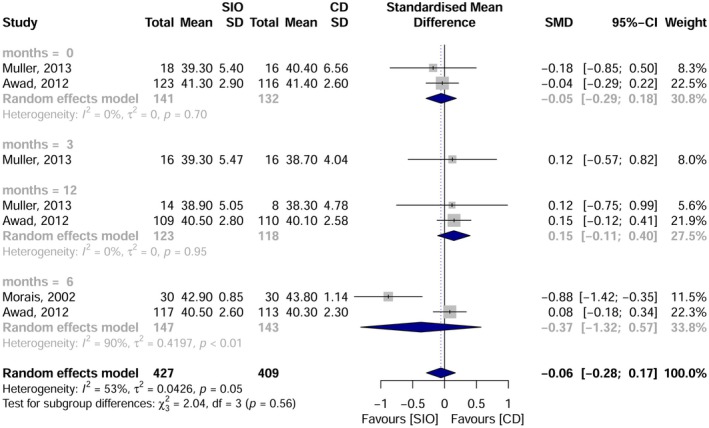
Meta‐analysis results for albumin.

**FIGURE 7:**
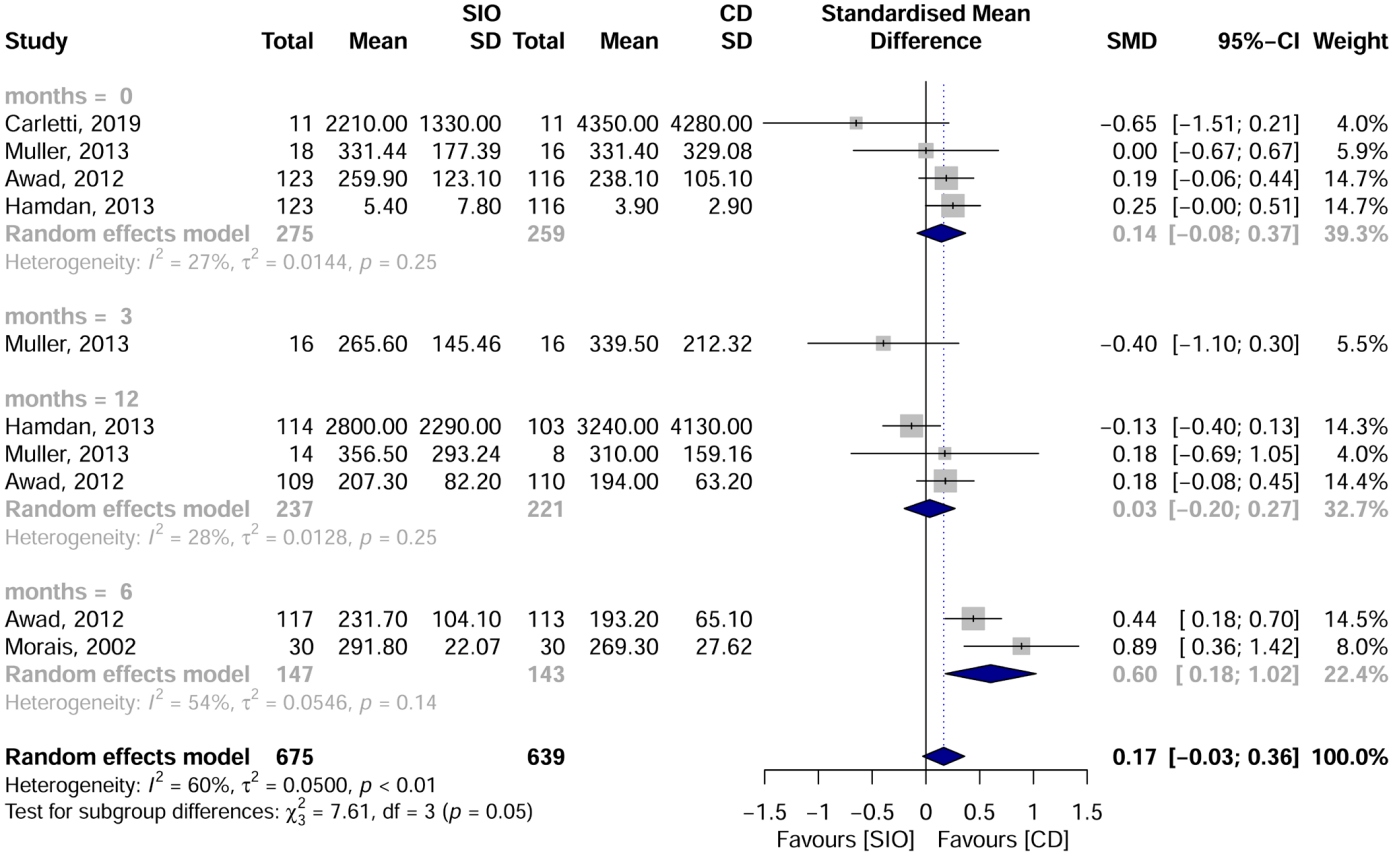
Meta‐analysis results for folate.

Five studies were included in the meta‐analysis, focusing on the intake of vitamin B12, folate and albumin at various time points: pre‐intervention, and at 3, 6 and 12 months post‐rehabilitation. Vitamin B12: No significant overall difference was observed. However, at 6 months, overdenture users had higher B12 levels than those using complete dentures [SMD = 0.60; 95% CI: 0.18–1.02; I^2^ = 54%] (Figure 5). Folate: No statistically significant difference between groups [SMD = −0.06; 95% CI: −0.28–0.17; I^2^ = 53%] (Figure 6). Albumin: No significant difference was detected [SMD = −0.09; 95% CI: −0.86–0.69; I^2^ = 98%] (Figure 7).

### 3.7 Certainty of the Evidence

The certainty of evidence, assessed using the GRADE framework, was rated as: Moderate for outcomes related to vitamin B12 and albumin; Low for folate.

These ratings reflect methodological limitations such as moderate risk of bias (e.g., inadequate randomisation); inconsistency due to variation in assessment protocols; imprecision related to small sample sizes and wide confidence intervals.

No publication bias was identified, as comprehensive efforts were made to include all relevant sources, including grey literature. None of the included studies reported conflicts of interest. Four studies [13, 14, 15, 17] were excluded from the GRADE assessment because they were not included in the quantitative synthesis (Table 2).

**TABLE 2:** Evidence synthesis.

Certainty assessment							No of patients		Effect		Certainty	Importance
No of studies	Study design	Risk of bias	Inconsistency	Indirectness	Imprecision	Other considerations	Overdenture	Prótese total	Relative (95% CI)	Absolute (95% CI)		
B12 Vitamin												
5	RCT	Not serious^a^	Not serious	Not serious	Not serious	None	635	639	—	SMD **0.17 SD higher** (0.03 lower to 0.36 higher)	⨁⨁⨁⨁ High^a^	Important
Albumine												
3	RCT	Not serious^b^	Not serious	Not serious	Not serious	None	427	409	—	SMD **0.06 SD lower** (0.28 lower to 0.17 higher)	⨁⨁⨁⨁ High^b^	Important
Folate												
3	RCT	Not serious^c^	Very serious^d^	Not serious	Not serious	None	618	588	—	SMD **0.09 SD lower** (0.86 lower to 0.69 higher)	⨁⨁◯◯ Low^c^, ^d^	Important

Abbreviations: CI, Confidence interval; RCT, randomised clinical trial; SMD, Standardised mean difference.

^a^ Three studies with moderate risk of bias.

^b^ Two studies with moderate risk of bias.

^c^ All studies with moderate risk of bias.

^d^ Sample heterogeneity.

## 4 Discussion

Edentulism is a multifactorial condition commonly associated with advancing age, smoking, periodontal disease and dental caries. Socioeconomic determinants such as low income, limited education, restricted access to dental care and systemic health inequalities further contribute to its prevalence [21, 22]. Prosthetic rehabilitation for edentulous patients typically involves either conventional complete dentures or implant‐supported overdentures (IODs) [7]. While IODs require surgical intervention—a factor that may discourage older individuals—and entail higher costs, they provide superior prosthesis retention, improved patient satisfaction and reduced alveolar bone resorption [7]. In contrast, conventional dentures are more accessible and non‐invasive but often lack functional stability, impairing the ability to chew hard or fibrous foods such as meats and raw vegetables [7].

This systematic review aimed to explore whether these different prosthetic modalities are associated with distinct nutritional outcomes in older adults. The findings suggest that overdenture users may exhibit better nutritional profiles in specific contexts, but the evidence remains inconclusive and heterogeneous.

The relationship between edentulism and overall health is well established. Tooth loss has been linked to an increased risk of cognitive disorders such as Alzheimer's disease and dementia, as well as to cardiovascular events [2, 4]. Given that adequate nutrition is a key determinant of frailty and comorbidity in older adults [16, 23, 24], maintaining a nutrient‐rich diet is essential for preserving systemic health and quality of life. Oral rehabilitation may play a crucial role in enabling older individuals to adhere to such diets [5, 6, 13, 23]. However, in the present review, quantitative analyses were limited to three biochemical markers—vitamin B12, folate and albumin—with statistically significant improvement observed only in vitamin B12 levels at six months among overdenture users.

These limited biochemical differences raise questions about behavioural and psychological factors influencing dietary patterns. It has been reported that psychosocial variables, such as mental health status and food‐related behaviours, significantly affect food choices in older adults [14]. As such, even when mechanical function is improved via overdentures, nutritional outcomes may remain unchanged without accompanying dietary counselling or behavioural support. The time‐dependent nature of habit formation may also explain why differences in vitamin B12 levels emerged only at six months, highlighting the potential for delayed nutritional improvements following prosthetic rehabilitation.

This limited biochemical response highlights a fundamental concept in geriatric rehabilitation: enhancing functional capacity does not guarantee behavioural change. While overdentures may improve masticatory efficiency, this mechanical improvement does not automatically translate into healthier dietary choices, particularly when ingrained food preferences and habits persist [25]. This gap between function and behaviour underscores the vital importance of nutritional counselling as an adjunct to prosthetic therapy [26]. A truly effective intervention likely requires an interdisciplinary approach, where dietary guidance helps patients leverage their restored masticatory ability to achieve meaningful nutritional improvements [27]. Consequently, the general lack of integrated nutritional counselling in the studies reviewed here may be a primary reason for the inconclusive evidence, suggesting a crucial direction for future clinical trial designs.

The individual study findings revealed conflicting patterns. For instance, Muller et al. [20] and El Osta et al. [15] observed improved anthropometric indicators in the overdenture group, while Gjengedal et al. [17] reported no significant differences. Carletti et al. [6] noted increased B2 and B6 levels in the overdenture group, whereas other studies [16, 18] identified higher vitamin and folate levels in complete denture users. Interestingly, a consistent finding across two studies [6, 13] was a reduction in sodium intake among overdenture users—an important result given the high cardiovascular risk associated with sodium excess in this age group.

From a broader perspective, overdenture users generally demonstrated better nutritional status and lower malnutrition risk [14, 15]. This may be partially attributed to improved masticatory ability, which facilitates the consumption of harder and more fibrous foods [16, 17, 19]. Nevertheless, it is noteworthy that even with functional improvements, many patients did not significantly alter their dietary habits [6, 15]. This stagnation could be explained by psychological barriers [14] or limited nutritional literacy, which may prevent effective dietary change [6, 15]. These findings emphasise the need for an interdisciplinary approach to oral rehabilitation that integrates nutritional guidance and individualised patient education alongside prosthetic treatment.

Several limitations must be acknowledged. First, the majority of included studies lacked long‐term follow‐up, limiting the ability to assess sustained changes in dietary behaviour or nutritional status. Second, the inclusion of observational studies introduces inherent risks of bias due to uncontrolled confounding variables. Third, the methodological quality of some randomised trials was compromised by incomplete reporting of randomisation and blinding procedures. Additionally, heterogeneity in outcome measures—including variation in nutrient assessment methods and time points—impeded direct comparison and robust pooling of results.

Despite these limitations, this systematic review provides a comprehensive and up‐to‐date synthesis of the literature on prosthetic rehabilitation and nutrition in older edentulous individuals. The findings offer valuable clinical insights and underscore the importance of combining prosthetic interventions with supportive behavioural and nutritional strategies to optimise systemic health outcomes.

## 5 Conclusion

Although implant‐supported overdentures are associated with improved masticatory efficiency and higher patient satisfaction, the current evidence does not support a consistent impact on nutritional intake when compared to conventional complete dentures. No significant differences were identified in key biochemical markers such as vitamin B12, albumin and folate. Given the limited and heterogeneous nature of the available data, it remains inconclusive whether overdentures confer superior nutritional benefits over conventional prostheses. Further high‐quality, long‐term studies are needed to clarify the systemic nutritional implications of different prosthetic rehabilitation approaches in older edentulous populations.

## Funding

The authors have nothing to report.

## Ethics Statement

The authors have nothing to report.

## Consent

The authors have nothing to report.

## Conflicts of Interest

The authors declare no conflicts of interest.

## Supporting information


**Appendix S1:** Supporting Information.

## Data Availability

All data supporting the findings of this study are included within the article and its supporting information.
