# Supplementary material for: Nutritional Outcomes of Overdentures vs. Complete Dentures in Older Edentulous Adults: A Systematic Review and Meta‐Analysis
Source: J Oral Rehabil. 2025 Dec 16;53(2):592–602. doi: 10.1111/joor.70111 (PMC12813523; doi:10.1111/joor.70111)
Supplement: Supplementary file 1 — Appendix S1: Supporting Information. [file JOOR-53-592-s001.docx]

**Appendix 1 –** Research words.

| Database | Search Aug, 16, 2023 / updated in June 2025 |
| --- | --- |
| Embase | ('overdenture':ti,ab,kw OR 'denture, overlay':ti,ab,kw OR 'dentures':ti,ab,kw OR 'ovelay denture':ti,ab,kw OR 'upper complete denture':ti,ab,kw OR 'lower complete denture':ti,ab,kw OR 'complete denture':ti,ab,kw OR 'overlay dentures':ti,ab,kw OR 'overdentures':ti,ab,kw OR 'hybrid prosthesis':ti,ab,kw OR 'telescopic prosthesis':ti,ab,kw OR 'dental prosthesis coating':ti,ab,kw OR 'prosthesis retention':ti,ab,kw OR 'prosthesis fixation':ti,ab,kw) AND ('nutrition':ti,ab,kw OR 'elderly nutrition':ti,ab,kw OR 'nutricional status':ti,ab,kw OR 'nutricional sciences':ti,ab,kw OR 'elderly nutrition physiological':ti,ab,kw OR 'phenomena':ti,ab,kw OR 'aged nutrition':ti,ab,kw OR 'aged nutrition physioly':ti,ab,kw) |
| Lilacs | ("overdenture" OR "Prótese Total" OR "Prótesis Sobredentadura" OR "prothèse Surdentaire" OR "Denture  Overlay" OR "próteses de sobreposição" OR "prothèse en recouvrement" OR "dentures" OR "próteses" OR "prothèses" OR "overlay denture" OR "prótese sobrepôs" OR "prothèse en recouvrement" OR "upper complete denture" OR "prótese completa superior" OR "prothèse complète supérieure" OR "lower complete denture" OR "prótese completa inferior" OR "prothèse complète inférieure" OR "complete denture" OR "prótese completa" OR "prothèse complète" OR "overlay dentures" OR "próteses de sobreposição" OR "prothèses de recouvrement" OR "overdentures" OR "sobredentaduras" OR "surdentures" OR "Hybrid Prosthesis" OR "Prótese Híbrida" OR "Prothèse Hybride" OR "Telescopic Prosthesis" OR "Prótese Telescópica" OR "Prothèse Télescopique" OR "Dental Prosthesis Coating" OR "Revestimento de Prótese Dentária" OR "Revêtement de Prothèse Dentaire" OR "Prosthesis Retention" OR "Retenção de Prótese" OR "Rétention de Prothèse" OR "Prosthesis Fixation" OR "Fixação de Prótese" OR "Fixation de Prothèse") AND ("Nutrition" OR "Nutrição" OR "Nutrición" OR "Nutrition" OR "Elderly Nutrition" OR "Nutrição de Idosos" OR "Nutrición de Personas Mayores" OR "Nutrition des Personnes Âgées" OR "Nutricional Status" OR "Estado Nutricional" OR "Estado Nutricional" OR "Statut Nutritionnel" OR "Nutricional Sciences" OR "Ciências Nutricionais" OR "Ciencias Nutricionales" OR "Sciences de la Nutrition" OR "Elderly Nutrition Physiological Phenomena" OR "Fenômenos Fisiológicos da Nutrição de Idosos" OR "Fenómenos Fisiológicos de Nutrición en Personas Mayores" OR "Phénomènes Physiologiques de Nutrition chez les Personnes Âgées" OR "Aged Nutrition" OR "Nutrição em Idosos" OR "Nutrición en Ancianos" OR "Nutrition des Personnes Âgées" OR "Aged Nutrition Physioly" OR "Fisiologia da Nutrição em Idosos" OR "Fisiología de la Nutrición en Ancianos" OR "Physiologie de la Nutrition chez les Personnes Âgées") |
| Livivo | TI=("overdenture" OR "Denture, Overlay" OR "dentures" OR "ovelay denture" OR "upper complete denture" OR "lower complete denture" OR "complete denture" OR "Overlay dentures" OR "overdentures" OR "Hybrid Prosthesis" OR "Telescopic Prosthesis" OR "Dental Prosthesis Coating" OR "[Prosthesis Retention](https://www.ncbi.nlm.nih.gov/mesh/68060489)" OR "Prosthesis Fixation") AND TI=("Nutrition" OR "Elderly Nutrition" OR "Nutricional Status" OR "Nutricional Sciences" OR "Elderly Nutrition Physiological" OR "Phenomena" OR "Aged Nutrition" OR "Aged Nutrition Physioly") |
| Pubmed | 1. ("overdenture"[MeSH Terms] OR "Denture, Overlay"[MeSH Terms] OR "dentures"[MeSH Terms] OR "ovelay denture"[MeSH Terms] OR "Denture, Complete, upper"[MeSH Terms] OR "Denture, complete, lower"[MeSH Terms] OR "Denture, complete"[MeSH Terms] OR "overdenture" OR "Denture, Overlay" OR "dentures" OR "ovelay denture" OR "upper complete denture" OR "lower complete denture" OR "complete denture" OR "Overlay dentures" OR "overdentures" OR "Hybrid Prosthesis" OR "Telescopic Prosthesis" OR "Dental Prosthesis Coating" OR "[Prosthesis Retention](https://www.ncbi.nlm.nih.gov/mesh/68060489)" OR "Prosthesis Fixation") 2. ("Nutrition"[MeSH Terms] OR "Elderly Nutrition"[MeSH Terms] OR "Nutricional Status"[MeSH Terms] OR "Nutricional Sciences"[MeSH Terms] OR "Nutrition" OR "Elderly Nutrition" OR "Nutricional Status" OR "Nutricional Sciences" OR "Elderly Nutrition Physiological" OR "Phenomena" OR "Aged Nutrition" OR "Aged Nutrition Physioly") 3. 1# AND 2# |
| Scopus | ("overdenture" OR "Denture, Overlay" OR "dentures" OR "ovelay denture" OR "upper complete denture" OR "lower complete denture" OR "complete denture" OR "Overlay dentures" OR "overdentures" OR "Hybrid Prosthesis" OR "Telescopic Prosthesis" OR "Dental Prosthesis Coating" OR "[Prosthesis Retention](https://www.ncbi.nlm.nih.gov/mesh/68060489)" OR "Prosthesis Fixation") AND ("Nutrition" OR "Elderly Nutrition" OR "Nutricional Status" OR "Nutricional Sciences" OR "Elderly Nutrition Physiological" OR "Phenomena" OR "Aged Nutrition" OR "Aged Nutrition Physioly") |
| Web of Science | 1. ("overdenture" OR "Denture, Overlay" OR "dentures" OR "ovelay denture" OR "upper complete denture" OR "lower complete denture" OR "complete denture" OR "Overlay dentures" OR "overdentures" OR "Hybrid Prosthesis" OR "Telescopic Prosthesis" OR "Dental Prosthesis Coating" OR "[Prosthesis Retention](https://www.ncbi.nlm.nih.gov/mesh/68060489)" OR "Prosthesis Fixation") 2. ("Nutrition" OR "Elderly Nutrition" OR "Nutricional Status" OR "Nutricional Sciences" OR "Elderly Nutrition Physiological" OR "Phenomena" OR "Aged Nutrition" OR "Aged Nutrition Physioly") 3. 1# AND 2# |
| Google Scholar | ("overdenture" OR "dentures") AND ("Nutrition") |
| Proquest | ("overdenture" OR "Denture, Overlay" OR "dentures" OR "ovelay denture" OR "upper complete denture" OR "lower complete denture" OR "complete denture" OR "Overlay dentures" OR "overdentures" OR "Hybrid Prosthesis" OR "Telescopic Prosthesis" OR "Dental Prosthesis Coating" OR "[Prosthesis Retention](https://www.ncbi.nlm.nih.gov/mesh/68060489)" OR "Prosthesis Fixation") AND ("Nutrition" OR "Elderly Nutrition" OR "Nutricional Status" OR "Nutricional Sciences" OR "Elderly Nutrition Physiological" OR "Phenomena" OR "Aged Nutrition" OR "Aged Nutrition Physioly") |

**Appendix 2 –** Excluded articles and reasons for exclusion.

| **Author, Year** | **Reason for exclusion** |
| --- | --- |
| ANDREAS ZENTHOfFER, A..; et al.; 2015 | 1,2 |
| BAXTER, J.C; et al.; 1983 | 6 |
| BERRETIN-FELIX, G; et al.; 2017 | 2 |
| BORGES T.D; et al.; 2011 | 3 |
| CHOY,Y; et al.; 2014 | 2 |
| ELWOOD, P.C; et al.; 1972 | 6 |
| MULLER, K; et al.; 2008 | 3 |
| OLIVEIRA, et al; 2001 | 6 |
| KAPUR, KRISHAN K; et al. 1998 | 3 |

**Legends**

1- Partially edentulous patients, removable partial dentures or no denture use.

2- Studies that did not compare removable total dentures with overdentures.

3- Patients under 60 years old.

4- Studies that did not evaluate the nutritional intake outcome.

5- Non-randomized studies, pseudo-randomized studies, reviews, expert opinions, case reports or conference abstracts.

6- The study was not found in any database, or google scholar, or grey literature.
